# Micrometre-scale deformation observations reveal fundamental controls on geological rifting

**DOI:** 10.1038/srep36676

**Published:** 2016-11-09

**Authors:** Johannes Thun, Ivan Lokmer, Christopher J. Bean, Eva P. S. Eibl, Bergur H. Bergsson, Aoife Braiden

**Affiliations:** 1 Geophysics Section, School of Cosmic Physics, Dublin Institute for Advanced Studies, Dublin, Ireland; 2 School of Earth Sciences, University College Dublin, Dublin, Ireland; 3 Icelandic Meteorological Office, Reykjavík, Iceland; 4 Research Management & Logistics Ltd, Dublin, Ireland

## Abstract

Many of the world’s largest volcanic eruptions are associated with geological rifting where major fractures open at the Earth’s surface, yet fundamental controls on the near-surface response to the rifting process are lacking. New high resolution observations gleaned from seismometer data during the 2014 Bárðarbunga basaltic dyke intrusion in Iceland allow us unprecedented access to the associated graben formation process on both sub-second and micrometre scales. We find that what appears as quasi steady-state near-surface rifting on lower resolution GPS observation comprises discrete staccato-like deformation steps as the upper crust unzips through repetitive low magnitude (*M*_W_ < 0) failures on fracture patches estimated between 300 m^2^ and 1200 m^2^ in size. Stress drops for these events are one to two orders of magnitude smaller than expected for tectonic earthquakes, demonstrating that the uppermost crust in the rift zone is exceptionally weak.

Ground deformation caused by magma migration and tectonic processes can often be observed in volcanic environments with ground- and satellite-based methods such as GPS and InSAR (e.g. refs 1, 2, 3, 4). The technical restrictions of these methods limit the smallest observable deformations to a few millimetres at best, with actual resolutions typically in the centimetre range^4^,^5^. For InSAR, the temporal resolution is further limited to several days. As a consequence, neither method currently allows us to investigate the micrometre scale nature of near-surface deformation processes. Instead we observe accumulated deformations that smooth out the underlying details of the ground deformation process. Hence the details regarding precisely how the Earth’s surface rifts at small spatio-temporal scales in volcanic environments are unclear.

An exceptional opportunity to investigate such deformation processes was posed by the 2014–2015 rifting episode and eruption at Bárðarbunga in Iceland, where we acquired data in the immediate vicinity of active surface rifting. Starting in August 2014, a lateral dyke propagated below the surface for over 45 km (Fig. 1a), indicated by the temporal and spatial evolution of seismicity and surface deformation patterns^4^,^6^. Interestingly, despite the high level of observed seismicity below a depth of about 3 km, there was a lack of shallow (<3 km deep) earthquakes associated with such a large rifting event^4^,^7^,^8^. The dyke eventually fed an eruption at the Holuhraun eruptive fissure, the southernmost tip of which was located approximately 5 km north of the Vatnajökull glacier rim. The effusive activity lasted for 4 h on 29 August 2014 and later continued for 6 months from 31 August 2014. In the area not covered by the glacier, divergent rifting (total surface opening ~2.5 m between mid-August and mid-September^4^,^9^) was observed at the surface, accompanied by substantial graben subsidence (2.5–5.5 m) directly above the inferred dyke^4^,^9^,^8^. The graben formation caused large surface fractures along its borders, revealed by satellite, aerial and field observations^8^,^9^ and the dip of the associated normal faults was estimated to be ~75°, based on the measured surface deformation^9^. In the northernmost region of the glacier, the graben formation caused an elongated dent in the relatively thin ice sheet^10^.

The details of the 2014–2015 Bárðarbunga volcano-tectonic episode have been addressed in numerous studies, e.g. refs 4, 6, 7, 8, 9, 10, 11. It was one of the largest rifting events and the largest effusive lava eruption in Iceland since the 1783–84 Laki eruption^12^ and offered an unprecedented opportunity to study rifting processes in detail.

## Experiment and Data Analysis

**Figure 1:**
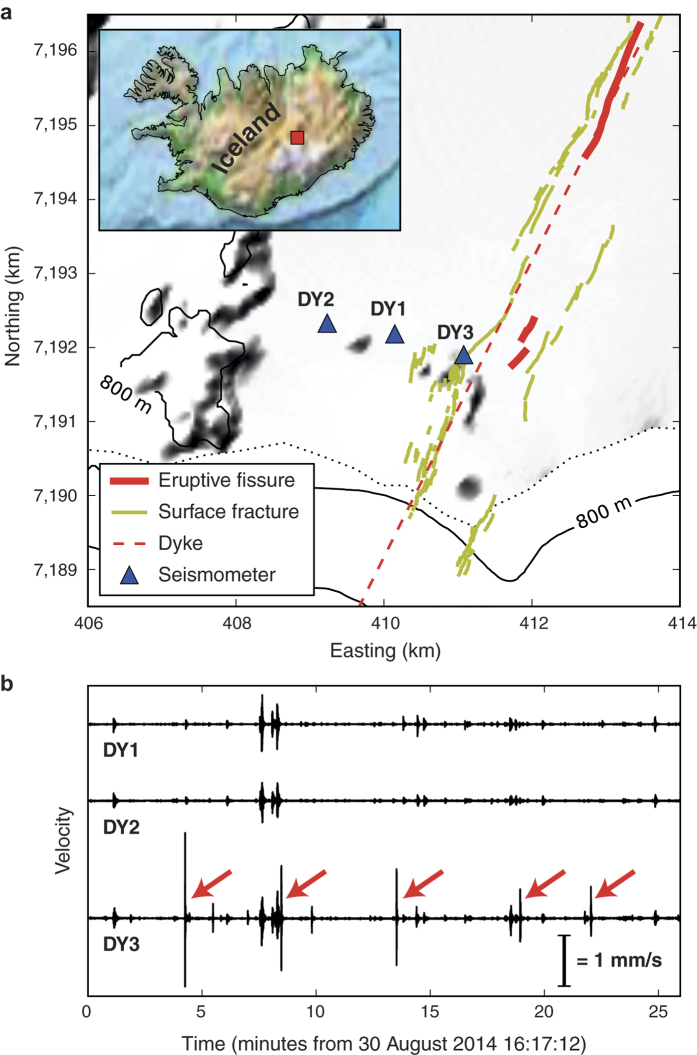
Overview of the seismic experiment and recorded data. (**a**) Map of stations DY1, DY2 and DY3 (Guralp 6TD seismometers) installed north of the Vatnajökull glacier (white) on 30 August 2014 immediately adjacent to several large surface fractures; fractures (yellow) and eruptive fissures (red) mapped by Hjartardòttir *et al*.^9^; dyke location (red dashed) inferred by Sigmundsson *et al*.^4^; elevation data from National Land Survey of Iceland. Inset map shows the location within Iceland (plotted with Matplotlib Basemap Toolkit^36^ using the ETOPO1 model^37^). (**b**) Unfiltered vertical recordings on all three stations. Arrows mark the step events on DY3 investigated in this study. These events are not visible on stations DY1 and DY2.

In the afternoon of 30 August 2014, we installed a small profile of three 3-component broadband seismometers (Guralp 6TD 30 s) perpendicular to the graben and inferred dyke (Fig. 1a), with the closest station (DY3) directly at the western shoulder of one of the large graben boundary faults and the other two stations approximately 1 km (DY1) and 2 km (DY2) from DY3. The surrounding area was characterised by several metres of poorly consolidated volcanic ash and sand on top of partially fractured basaltic lava flows^13^, a strongly scattering environment for seismic waves. As strong ground shaking could be felt during the experiment, the operation had to be aborted for safety reasons, resulting in ~26 minutes of synchronous data on all stations. On 5 September 2014, a new fissure opened approximately 600 m east of DY3 and effused lava for 2 days.

**Figure 2:**
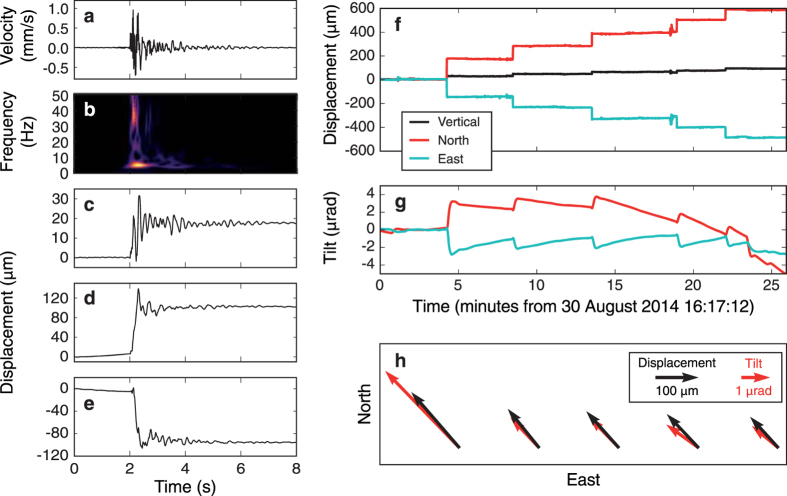
Individual step event (**a–e**) and processed full-length seismograms showing ground deformation (**f–h**) at station DY3. (**a**) Vertical velocity waveform (instrument corrected). (**b**) Scalogram illustrating relative frequency content – the main frequency peak lies between 3 Hz and 8 Hz, with an additional peak above 25 Hz. (**c–e**) Vertical, North and East displacements, respectively; processed with the median filter method^14^. The resulting displacement step is about 17 μm upward and 123 μm in a northwest direction. (**f**) Median filter processed seismogram for step recovery (black: Vertical, red: North, cyan: East), showing a consistency of step direction for the individual events. Note that between events 3 and 4, a longer period event impedes the filter performance, leading to a slight artificial step. (**g**) Tilt record retrieved from seismograms using the tilt transfer function^18^. While the long-period trend is not interpreted here, each of the 5 events shows a clear tilt step on both horizontal components. (**h**) Directions and amplitudes of horizontal deformations of the 5 steps seen in (**f**,**g)**.

The unprocessed vertical velocity seismograms (Fig. 1b) show coherent activity on all three stations. However, the focus of this study lies in five high amplitude signals on station DY3 (red arrows in Fig. 1b), which are not registered by the other stations DY1 and DY2, suggesting that the causative events are relatively small and local to DY3. The velocity seismograms and the corresponding scalograms of these events (event number 3 shown in Fig. 2a,b) show impulsive waveforms with a main frequency peak between 3 and 8 Hz and a secondary peak above 25 Hz. As high-frequency waves are attenuated strongly when travelling through the ground, such high frequencies thus indicate a fracturing process close to the station. In a recent study^14^, we presented a new data processing approach that allows for the recovery of micrometre-scale displacement steps from instrument-corrected seismograms. It is based on long-period noise removal using median filters and its performance was confirmed by laboratory experiments. We apply this method to the event (Fig. 2c–e) and observe displacement steps on all three (orthogonal) components of the instrument, i.e. the station was displaced by approximately 125 μm in a northwest, slightly upward direction. This represents a motion away from the centre of the graben and the underlying dyke. Applying this procedure to the full-length records on this station reveals similar amplitudes and ratios between different displacement components for all five events (Fig. 2f), suggesting a repetitive process with similar source locations and an apparent average inter-event time of about 4.5 minutes. As horizontal components of seismometers are also susceptible to ground rotation^15^,^16^,^17^, possible tilts can be estimated from the data using the tilt transfer function^18^,^19^,^20^; this involves a simple integration of raw data and multiplication with a factor depending on well-known instrument properties. The resulting traces (Fig. 2g) show tilt steps of 1.3–4 μrad oriented in a northwest direction associated with each of the five events. The tilt step directions coincide with the direction of the displacement steps (Fig. 2h) and support a repeating source process generating the events, with roughly consistent amplitudes and locations.

### Source location

Although one station is not sufficient to fully invert for source locations and mechanisms, we use the observed static deformations from DY3 to explore potential sources with a forward modelling approach. We estimate the source location and magnitude by (i) assuming a plausible source mechanism and (ii) performing a search over a 200 × 200 × 100 m^3^ grid around the station, where we match the observed ratios between different deformation components with the theoretical values for a homogeneous, elastic medium^21^. The ratios are defined as


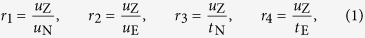


where *u*_Z_, *u*_N_ and *u*_E_ are displacements and *t*_N_ and *t*_E_ are tilts. Subscripts Z, N and E denote a vertical, north and east direction, respectively. These ratios are used to compute two misfits, *R*_d_ (displacements only) and *R*_dt_ (both displacements and tilts), defined as:


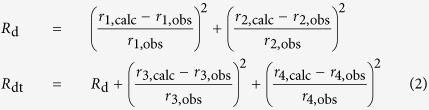


The minimum misfits indicate the best location for the chosen source mechanism and the corresponding seismic moment *M*_0_ can be found by a simple least squares inversion.

**Figure 3:**
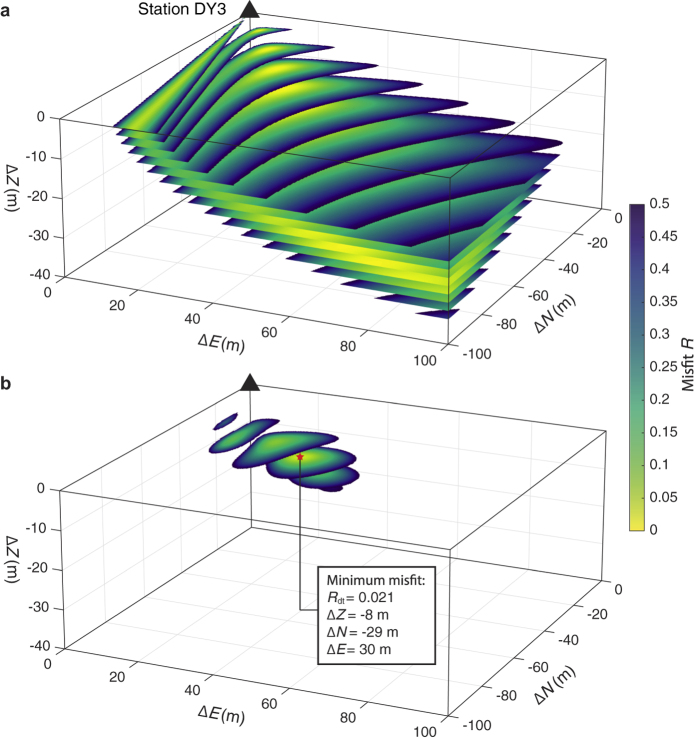
Misfits between field observations and displacements and tilts calculated using analytical solutions^21^ for a 75° dip-slip (normal fault) source. (**a**) Misfit *R*_d_ using only displacement ratios. (**b**) Misfit *R*_dt_ using both displacement and tilt ratios, showing a single confined minimum at Δ*Z* = −8 m, Δ*N* = −29 m, Δ*E* = 30 m. Misfits are displayed in horizontal slices of 2 m spacing and values above *R* = 0.5 are not shown. Material parameters for both (**a**,**b)** *V*_P_ = 500 m/s and *ν* = 0.3.

As the station is located in direct proximity to the faults associated with the graben formation, we suspect that the local events are part of the faulting process. Consequently, we assume a 75° dip-slip mechanism parallel to the N25°E striking boundary fault^9^ as the source mechanism. Figure 3 shows the misfits for this normal fault mechanism, where the observed tilt and displacement values of the third step (Fig. 2f,g) are used. Here we assume a medium P-wave velocity of *V*_P_ = 500 m/s and Poisson’s ratio of *v* = 0.3, consistent with values obtained for unconsolidated upper geological layers at various volcanoes^22^,^23^,^24^,^25^,^26^ (further discussion in supplementary information). For clarity, only misfit values below 0.5 are displayed and all remaining misfits are located in the quadrant south-east of the source. As the displacement-only misfit *R*_d_ (Fig. 3a) does not converge around a single minimum, it can only indicate the approximate direction of the source with respect to DY3. When tilts are introduced (Fig. 3b), a sharp minimum misfit *R*_dt_ is found approximately 40 m southeast of DY3 at a depth of 8 m. The best-fitting source moment at this location is *M*_0_ = 1.5 × 10^8^ Nm (moment magnitude *M*_W_ = −0.6). When we change *V*_P_ to 1000 m/s, the source location remains unchanged, with the source moment increasing to *M*_0_ = 7.1 × 10^8^ Nm (*M*_W_ = −0.2). The same grid search with different medium parameters leads to similar source-receiver distances and source moments (Supplementary Table 1). The source moments are small enough to justify the use of the point-source assumption in our forward modelling approach.

Static displacements such as those observed at DY3 are near- and intermediate-field effects and can only be observed within a fraction of a wavelength from the seismic source^27^. The sources inferred above would theoretically cause total static displacements smaller than 1 µm at the next closest station, DY1. Sub-micrometre steps are not detectable with our instruments and methods^14^. The fact that the events are not visible at the other stations also implies that the dynamic seismic signals, i.e. all near-, intermediate- and far-field components, fall under the noise level at these locations, likely due to strong wave attenuation in the unconsolidated surface materials^13^.

### Source dimensions

**Figure 4:**
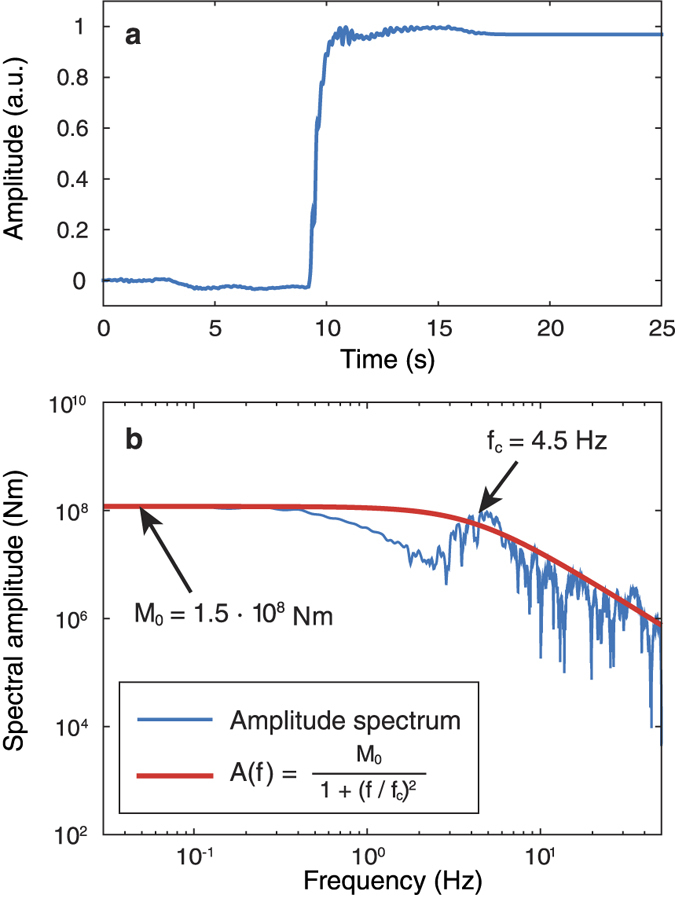
Source time-function and its amplitude spectrum. (**a**) Normalised source-time history (slip on the fault). (**b**) Moment-rate spectrum fit with a *ω*^2^-source model^28^. The flat part of the spectrum (left arrow) corresponds to the seismic moment *M*_0_, while the corner frequency *f*_c_ (right arrow) is related to the source size. The results shown are calculated for *V*_P_ = 500 m/s and *ν* = 0.3 and the source location from Fig. 3b. Note that varying *ν* between 0.25 and 0.35 does not affect the corner frequency to a large extent (*f*_c_ = 4.2–5.6 Hz).

For the source location found above, we determine source parameters (size and slip) by removing path effects through deconvolving modelled deformation and seismic radiation (Green’s function) from the recorded seismogram shown in Fig. 2c. Here we use the linear relationship between the ground displacement spectrum *U*(*ω*) and the source moment spectrum *M*(*ω*)^27^:


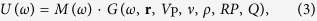


where the Green’s functions *G* depend on the receiver position ***r*** relative to the source, the elastic properties of the medium *V*_P_ and *ν*, the density *ρ*, the radiation pattern *RP* for a specific source mechanism and the quality factor *Q*. This simplifies the deconvolution to a simple division *M* = *U*/*G* for each frequency. The resulting source moment spectrum *M*(*ω*) is subsequently fit with a Brune *ω*^2^ source model^28^ in order to determine the corner frequency. We calculate *G*(*ω*) for the inferred normal fault source with the expressions given by Aki and Richards^29^ and modified by Lokmer and Bean^27^, using the same parameters as above (*V*_P_ = 500 m/s, *ν* = 0.3). *Q* is varied until we obtain the best fit to the *ω*^2^-model (*Q* = 20). The source-time history *M*(*t*) resulting from this deconvolution is shown in Fig. 4a. Its spectrum and the *ω*^2^-model fit are shown in Fig. 4b, resulting in a corner frequency of 4.5 Hz.

This frequency is used to determine the source size (and subsequently the slip *D* using *M*_0_ = *μAD*, with the shear modulus *μ* and the source area *A*): approximating the source as a slipping circular patch^30^,^31^ gives a source radius of approximately 10–20 m with an average slip of 1–4 mm. As the actual source mechanism cannot be inferred from our data and a tensile component could potentially form part of the source process, we additionally performed the location grid search and source slip analysis for a tensile crack (Supplementary Figure 1 and Supplementary Table 1). If a purely tensile source mechanism is considered, the slip displacement on the same patch is reduced by a factor of 2, showing the results are robust for a deviation from the pure normal faulting source. Both results are in agreement with Liu-Zeng *et al*.^32^, who model the slip-to-length ratio and obtain equivalent values for small faults with rough fault surfaces.

We estimate stress drops of Δ*σ* = 0.008–0.07 MPa using Δ*σ* = 7*M*_0_/16*r*^3^ according to Eshelby^33^. These values are 2–3 orders of magnitude smaller than expected for tectonic seismicity (stress drops typically >1 MPa^34^) and point to a very weak uppermost crust. They are consistent with the lack of shallow “standard” earthquakes associated with such a large rifting event. Such small stress drops are in striking agreement with the value of Δ*σ* = 0.01 MPa obtained for shallow long period seismicity on Mt Etna, Italy^35^, attributed to the presence of exceptionally weak near surface volcanic material that could not sustain high shear or tensile stresses and hence also failed at exceptionally low earthquakes magnitudes.

## Discussion

Our data reveal new information about the rifting process, suggesting that it is at least partially discrete, occurring in micrometre scale steps. This raises questions about how these displacements compare to the observed long-term deformation in the area. Combining the time-history of the closest GPS stations with the total graben opening measured from satellite data (see supplementary information), the deformation rate for 30 August 2014 is estimated to be roughly 5 cm/day. Assuming a repeating process with average displacement steps of 133 μm and average inter-event times of 267 s, observed at DY3, we extrapolate our data and obtain an approximate deformation rate of 4.3 cm/day. Furthermore, accumulating normal fault slip estimates at the source of 1–4 mm yields a horizontal deformation rate of 7–27 cm/day. Although the modelled slip values are approximate, both of our displacement measures are in good agreement with the GPS estimates. The similarity suggests that the satellite and GPS-derived long-term surface deformation associated with Earth surface rifting is a consequence of displacement accumulated through very low magnitude discrete brittle failure at the millimetre scale. The detection of such steps is limited to distances within a few hundred metres from the source, highlighting the rarity of such observations. The similarity also suggests that any aseismic component is small at the spatial and temporal scales captured in this study; it also indicates that fracturing of the weak uppermost crust is limited to microseismic events, consistent with the lack of observed shallow seismicity^4^,^7^.

We conclude that at its smallest temporal and spatial scales, rifting in the uppermost Earth’s crust is not a steady state process but rather exhibits transient staccato-like behaviour that yields definable spreading rates only when viewed over longer time scales. Stress drop analysis on the discrete micro-events reveals that the uppermost crust is exceptionally weak in the rift zone.

## Additional Information

**How to cite this article**: Thun, J. *et al*. Micrometre-scale deformation observations reveal fundamental controls on geological rifting. *Sci. Rep*. **6**, 36676; doi: 10.1038/srep36676 (2016).

**Publisher’s note:** Springer Nature remains neutral with regard to jurisdictional claims in published maps and institutional affiliations.

## Supplementary Material

Supplementary Information
