# Supplementary material for: Micrometre-scale deformation observations reveal fundamental controls on geological rifting
Source: Sci Rep. 2016 Nov 9;6:36676. doi: 10.1038/srep36676 (PMC5101494; doi:10.1038/srep36676)
Supplement: Supplementary Information [file srep36676-s1.doc]

**Micrometre-scale deformation observations reveal fundamental controls on geological rifting**

**J. Thun, I. Lokmer, C. J. Bean, E. P. S. Eibl, B. H. Bergsson, A. Braiden**

*Scientific Reports* **6**, 36676 (2016)

**Supplementary Information**

**Supplementary discussion: Material parameters and the influence on the forward modelling**

In order to calculate the displacements and tilts^21^, we need the Lamé parameters (*λ, μ*) and the density *ρ* (alternatively, the P-wave velocity *V*_p_ and Poisson’s ratio *ν* and the density). We estimate the density *ρ* from *V*_p_ using Gardner’s relation^39^, leaving only the two independent parameters *V*_p_ and *ν*.

**Poisson’s ratio *ν*:** Several seismic refraction profiles provide information on the structure and material parameters of basaltic lavas in the volcanic zone and tertiary regions in Northern and Eastern Iceland, although no information is available for the exact location of our experiment^40^. Poisson’s ratios are mostly found between 0.25 and 0.3 with very few values outside this range. The profiles closest to our experiment location (profile 18 in ref. 40) show ratios of just under 0.3, the value we chose for Fig. 3. Studies on other volcanoes found shallow Poisson’s ratios up to 0.35^22,23^, so we calculated the misfits for the ratios of 0.25, 0.3 and 0.35.

**P-wave velocity *V*_P_:** The variation of *V*_P_ between different profiles^40^ is much more pronounced for top layers, as the heterogeneous surface structures and materials can change drastically at short lateral distances. The average value for the topmost layer (130 m thickness) from the closest refraction profile where a top layer was resolved (profile 21 in ref. 40) is *V*_P_ = 2560 m/s. However, as the lava in the area of our experiment is covered by several meters of ash and sand, and we are dealing with very shallow sources, the effective velocities are most likely significantly lower. In studies investigating shallow velocity structures on various volcanoes, top layers of several to tens of meters with P wave velocities down to as low as 400 m/s were found and potentially attributed to unconsolidated materials^22-26^, similar to those which we encounter at the experiment site.

As we have little knowledge of the shallow structures and local rock parameters at the site of the experiment, we explored their influence on the locations and moments by performing our analysis for different velocities *V*_p_ of 500 m/s and 1000 m/s and Poisson’s ratios *ν* of 0.25, 0.3 and 0.35. The results (Supplementary Table 1) show that the minimum misfit locations shift when varying *ν*, by no more than ~4 m for a dip-slip mechanism and approximately 11 m for a tensile crack mechanism. Changing *V*_p_ only controls the obtained source moment in this setup (as expected from the equations^21^), with a velocity of 1000 m/s yielding moments about 5 times bigger than for *V*_p_ = 500 m/s.

**Supplementary discussion: Deformation rate estimate based on GPS and InSAR data**

Since there were no GPS sensors installed at our experiment site, we do not have direct measurements of the local surface deformation for 30 August 2014. An estimate for this can be obtained by combining the displacement history from the closest GPS stations with the local displacements obtained from InSAR and modelling work. At station GSIG, ~20 km southeast of DY3, the horizontal deformation rate on 30 August 2014 was about 15 mm/d (ref. 4, Supplementary Fig. 1d). The total southeast-displacement over the entire rifting event was about 360 mm at GSIG. Around seismometer station DY3, the total surface opening of the graben was about 2.5 m, i.e. 1.25 m of displacement on either side^4,9^. Assuming that the relative displacement history is the same for both locations, we obtain a deformation rate of (1250 mm / 360 mm) * 15 mm/d ≈ 52 mm/d. Nearly the same result is obtained when using GPS station DYNC (~25 km ESE of DY3) for the displacement history, so this is an approximate, yet robust estimate for the deformation rate at DY3 for the day of the experiment, which compares well with the value that we estimate from the seismometer data (43 mm/d, see main text).

**
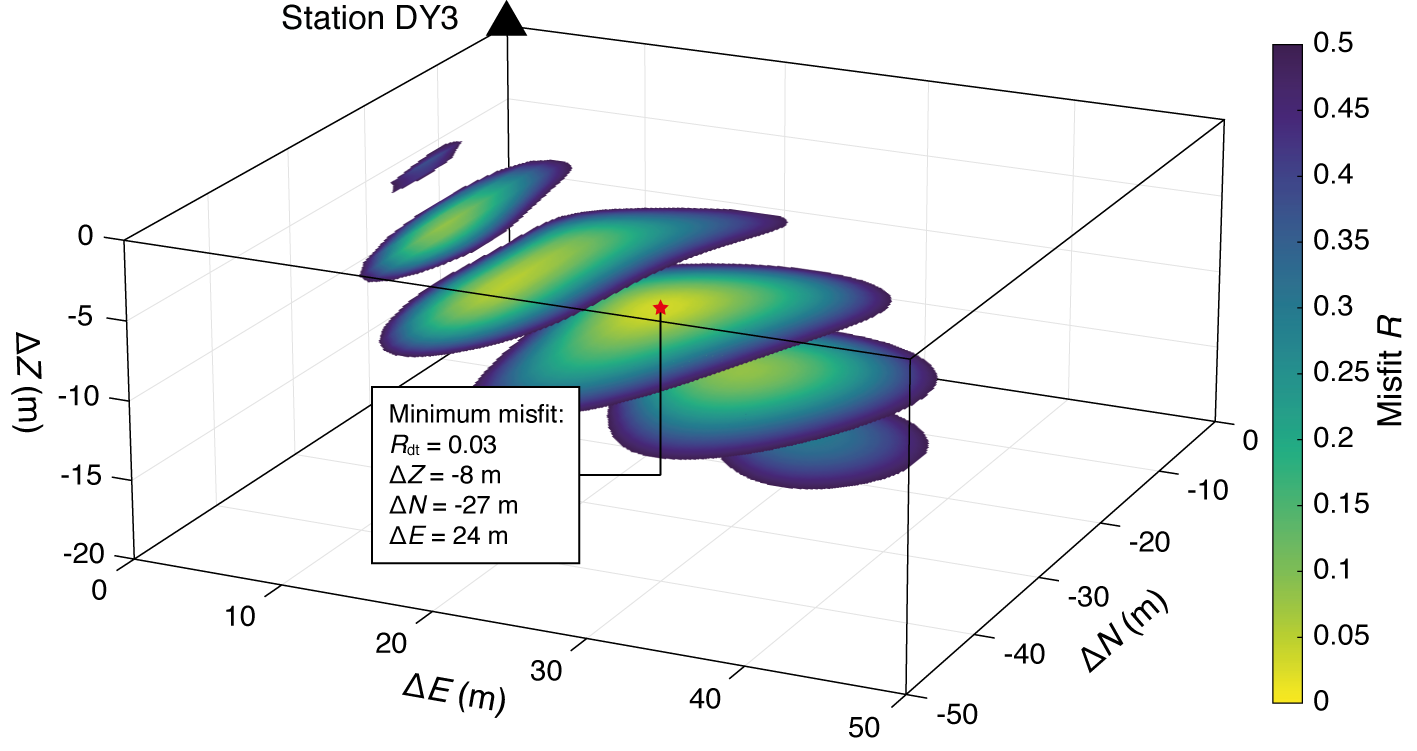
**

**Supplementary Figure 1 | Misfits *R*_dt_ between field observations and displacements and tilts calculated using analytical solutions^15^*.***

Solution for a tensile crack source parallel to the dyke segment striking at N25°E ^(4)^, minimum at Δ*Z* = -8 m, Δ*N* = -27 m, Δ*E* = 24 m. Misfits are displayed in horizontal slices with 2 m spacing and values above *R* = 0.5 are not shown. Material parameters: *V*_P_ = 500 m/s and *ν* = 0.3.

| **Mechanism** | ***ν*** | ***V*_P_ (m/s)** | **Δ*Z* (m)** | **Δ*N* (m)** | **Δ*E*(m)** | ***M*_0_ (Nm)** | ***R*_dt_** |
| --- | --- | --- | --- | --- | --- | --- | --- |
| **Dip-slip** | 0.25 | 500 | -8 | -31 | 26 | 1.8 · 10^8^ | 0.018 |
| ***θ* = 25°** |  | 1000 | ″ | ″ | ″ | 8.3 · 10^8^ | ″ |
| ***δ* = 75°** | 0.3 | 500 | -8 | -30 | 29 | 1.5 · 10^8^ | 0.021 |
|  |  | 1000 | ″ | ″ | ″ | 7.1 · 10^8^ | ″ |
|  | 0.35 | 500 | -7 | -28 | 28 | 1.1 · 10^8^ | 0.023 |
|  |  | 1000 | ″ | ″ | ″ | 5.2 · 10^8^ | ″ |
| **Tensile** | 0.25 | 500 | -7 | -33 | 16 | 8.9 · 10^7^ | 0.006 |
| ***θ* = 25°** |  | 1000 | ″ | ″ | ″ | 4.2 · 10^8^ | ″ |
| ***δ* = 90°** | 0.3 | 500 | -8 | -27 | 24 | 5.2 · 10^7^ | 0.03 |
|  |  | 1000 | ″ | ″ | ″ | 2.5 · 10^8^ | ″ |
|  | 0.35 | 500 | -7 | -25 | 24 | 3.6 · 10^7^ | 0.036 |
|  |  | 1000 | ″ | ″ | ″ | 1.7 · 10^8^ | ″ |

**Supplementary Table 1 | Minimum misfit solutions from the Okada grid search for different source mechanisms and medium parameters.**

*θ* indicates the strike angle (from north) and *δ* the dip angle (from horizontal) of the fault. Locations Δ*Z,* Δ*N* and Δ*E* are relative to station DY3.

**Additional references**

1. Gardner, G. H. F., Gardner, L. W. & Gregory, A. R. Formation velocity and density - the diagnostic basics for stratigraphic traps. *Geophysics* **39(6),** 770-780 (1974).
2. Pálmason, G. Seismic refraction investigation of the basalt lavas in northern and eastern Iceland. *Jökull* **13,** 40-60 (1963).
